# A multimodal approach for the ecological investigation of sustained attention: A pilot study

**DOI:** 10.3389/fnhum.2022.971314

**Published:** 2022-09-29

**Authors:** Keren Avirame, Noga Gshur, Reut Komemi, Lena Lipskaya-Velikovsky

**Affiliations:** ^1^Psychiatric Division, Sourasky Medical Center, Tel Aviv-Yafo, Israel; ^2^Independent Researcher, Tel Aviv-Yafo, Israel; ^3^School of Occupational Therapy, Faculty of Medicine, Hebrew University, Jerusalem, Israel

**Keywords:** one-channel EEG, attentional control, test of everyday attention, everyday functioning, attention fluctuations

## Abstract

Natural fluctuations in sustained attention can lead to attentional failures in everyday tasks and even dangerous incidences. These fluctuations depend on personal factors, as well as task characteristics. So far, our understanding of sustained attention is partly due to the common usage of laboratory setups and tasks, and the complex interplay between behavior and brain activity. The focus of the current study was thus to test the feasibility of applying a single-channel wireless EEG to monitor patterns of sustained attention during a set of ecological tasks. An EEG marker of attention (BEI—Brain Engagement Index) was continuously recorded from 42 healthy volunteers during auditory and visual tasks from the Test of Everyday Attention (TEA) and Trail Making Test (TMT). We found a descending pattern of both performance and BEI in the auditory tasks as task complexity increases, while the increase in performance and decrease in BEI on the visual task. In addition, patterns of BEI in the complex tasks were used to detect outliers and the optimal range of attention through exploratory models. The current study supports the feasibility of combined electrophysiological and neurocognitive investigation of sustained attention in ecological tasks yielding unique insights on patterns of sustained attention as a function of task modality and task complexity.

## Introduction

Sustained attention represents a basic attentional function and is defined as the ability to maintain attention and remain in a state of vigilance throughout an extended period (Esterman and Rothlein, 2019). Being a basic, yet complex, cognitive function, sustained attention encompasses a variety of functions, including information selection, enhancement of selected information, and inhibition of unselected information, thus allowing an appropriate response to infrequent and unpredictable stimuli (Petersen and Posner, 2012; Esterman and Rothlein, 2019). Importantly, sustained attention has a direct impact on the efficacy of additional aspects of attention (selective attention, divided attention), other cognitive skills (e.g., memory, executive functions), and daily living functions such as academic attainment, employment performance, and safe driving (Schmidt et al., 2009; Fortenbaugh et al., 2015; Tan and Thamarapani, 2019).

Sustained attention is a fluctuating function, possibly due to survival needs (Robertson and O’Connell, 2010). This innate property of sustained attention is often explained in light of theories of arousal, mind wandering, cognitive resource allocation, and effort (Shenhav et al., 2017; Esterman and Rothlein, 2019). However, fluctuations in sustained attention also derive from task characteristics such as stimulus modality (e.g., visual or auditory), density and intensity, task duration, novelty, and complexity (Petersen and Posner, 2012). For example, Szalma et al. (2004) showed that attentional decrement was less pronounced for auditory than for visual tasks and that recovery from a stressful experience occurs in auditory but not in visual conditions. Moreover, an increase in task complexity can lead to different patterns of attention allocation, such as an inverted U shape (He and Zempel, 2013) or linear patterns (Staal, 2004), emphasizing the importance of investigating the specific dynamic of sustained attention.

Sustained attention is commonly estimated through behavioral laboratory stimuli-based tasks. The most well-known instrument is the Continuous Performance Test (CPT, Egeland and Kovalik-Gran, 2010). So far, the ecological validity of the CPT was found to be inconsistent (Hall et al., 2016; Berger et al., 2017), further supporting the notion that sustained attention should be investigated through ecological tasks to better understand the high costs of attentional failures that contribute to road, railway, and aircraft incidences (Murphy et al., 2016). Indeed, most everyday tasks that require sustained attention bring together multiple dimensions of attention while evoking natural fluctuations which are hard to test in lab-based tasks such as CPT (Murphy et al., 2016). First steps toward the development and implementation of ecological assessments in healthy and clinical populations have been taken. For example, the classical go-no-go paradigm was implemented within the Test of Everyday Attention (TEA) through daily life ecological stimuli including tasks such as a visual map or telephone number searching while doing auditory tasks (Robertson et al., 1994).

Brain activity underlying sustained attention long has been studied with neuroimaging methods such as electroencephalogram (EEG). Traditionally, sustained attention was measured in the lab with high-density EEG devices recording spectral EEG or task-related EEG responses to assess cognitive functions. Given that traditional EEG paradigms are unsuitable for ecological setups, a recent body of work increasingly promotes the usage of real-time, easy-to-use EEG technologies in ecological research and clinical practice (Lau-Zhu et al., 2019). For example, it was demonstrated that EEG markers obtained during sustained attention tasks are sensitive to the parameters of everyday functioning, such as driving or job-load (Kamzanova et al., 2014; Rupp et al., 2018). Despite some methodological concerns (Rieiro et al., 2019), such as the use of a single-channel dry electrode, signal quality, and artifacts, researchers applied innovative models for analyzing the ongoing data to provide an automated simplified signal for state-dependent classification of the level of attentiveness (Liu et al., 2013), detection of moment-to-moment attention fluctuations during a computerized CPT (Zhang et al., 2021), and early identification of attentional problems in children (Serrano-Barroso et al., 2021). Similarly, the relatively new brain engagement index (BEI), offering an attention-related index, has been tested in diverse clinical populations such as stroke (Bartur et al., 2017), migraine (Shahaf et al., 2018a), and depression (Shahaf et al., 2017). In a recent study, BEI was applied during CPT before and after stimulant intake and was found to be practical in classifying subjects into healthy controls or ADHD (Shahaf et al., 2018b). As such, BEI and other EEG indexes have the potential to be repeatedly applied for real-life dynamic monitoring as opposed to the current standardized evaluation.

Thus, the current pilot study aimed at investigating patterns of brain activity and behavior in ecological tasks of sustained attention. For this purpose, we used the Test of Everyday Attention (TEA) since it contains tasks from everyday life and its validity and feasibility are well established (Robertson et al., 1996). The TEA consists of a set of short ecological tasks of visual and auditory modality with a gradual increase in complexity. The TEA was previously tested in a range of populations (van der Leeuw et al., 2017) and was found to correlate with community functioning (Tyson et al., 2008). We also controlled for cognitive level using the Trail Making Test (TMT) and tested its relationship with attentional functions (Gillen, 2008). In addition, we applied a consumer single-channel wireless EEG device (NeuroSky MindWave) during the TEA and TMT tests. We aimed at monitoring brain engagement (BEI) through recording of ongoing electrophysiological frontal activity which is directly computed to provide an online index of attention (Shahaf et al., 2018a). As such, we investigated, in a preliminary manner, a multimodal approach for studying patterns of sustained attention as a function of task modality and task complexity. We also analyzed the BEI indices in the complex tasks, in an exploratory manner, to detect outliers and optimal range of attention. We believe that the implementation of real-time EEG monitoring in this setup will expand our understanding of how to proceed in better assessing sustained attention in the context of real-world functioning.

## Materials and methods

### Participants

Forty-two healthy volunteers were recruited for the study through advertising on social media. The participants aged 18–48 years old (*M* = 25.9, *SD* = 4.8) were mostly female (*N* = 29, 69%), had at least 12 years of education (secondary school), no history of neurodevelopmental or acquired neuropsychiatric disorders, and use of neuroleptic or psychotropic drugs. All the participants had normal or corrected vision, normal hearing, did not take any medication affecting the level of arousal in the previous 24 h (e.g., analgesics), slept at least 6 h before study participation, and did not report unusual stress (24 h). The participants completed screening for cognitive impairment before entering the study and were found to be within the intact range (The Montreal Cognitive Assessment: 26–30, *M* = 28, *SD* = 1.6).

### Measures

#### Cognitive screening

The Montreal Cognitive Assessment (MoCA) (Nasreddine et al., 2005) is a paper-based screening instrument for the detection of mild cognitive impairment. The test is composed of eight parts and evaluates visuospatial perception, organizational skills, recognition and naming, short-term memory, attention, verbal ability, abstraction, and orientation. It takes about 10 min and produces a maximum possible score of 30 points. The cut-off of 26 points was set to mark normal cognitive function and was applied in this study as exclusion criteria (Pike et al., 2017).

#### Experimental cognitive tasks

Trail Making Test (TMT) (Gaudino et al., 1995) is a widely used, well-established neuropsychological tool to measure the cognitive domains of visual-motor processing speed, sequencing, and cognitive flexibility, as a part of attentional control. TMT comprises two parts: in part A, the participant is asked to connect a series of 25 encircled numbers in numerical order; in part B, the participant is asked to connect 25 encircled numbers and letters in numerical and alphabetical order, alternating between the numbers and letters in ascending order. The primary score of the test is the total time (seconds) to completion of parts A and B, including correction if required. In the current study, TMT was used to assess cognitive level (Bowie and Harvey, 2006) and its contribution to attentional functions was tested (Gillen, 2008).

The test of Everyday Attention (TEA) (Robertson et al., 1994) was designed based on the go-no-go paradigm, previously established for investigating attention while implementing daily life tasks and using stimuli of visual map searching, elevator-floor counting (visual and auditory), visual telephone number searching, telephone number searching while doing auditory tasks, and auditory lottery task. Altogether, the test consists of eight subtests designed to assess aspects of visual and auditory sustained attention, including vigilance to stimuli and attentional switches. Each subtest consists of 7 up to 10 trials with graduated levels of complexity in terms of stimulus load: number of distractors, types of stimuli, and the trial duration. For this study, we used “elevator tasks”: Three auditory tasks and one visual task. In each task, the participant is asked to count at which floor the elevator stopped based on auditory or visual information, each one with a gradual rise in the level of complexity as for the number of stimuli, their complexity, and the trial duration. The auditory tasks include tones counting for the floor identification as follows: 1st subtest—basic tones counting (one type, 7 trials); 2nd subtest—basic tones counting with distraction (10 trials); 3rd subtest—basic tones counting with distraction and mental manipulation (additional types of tones are added to this task to indicate the direction of the elevator movement: up or down with requirement for follow up the switches, 10 trials); and 4th subtest—visual elevator counting with mental manipulations to follow switches (up and down, random order) in the elevator movement directions (10 trials). The content and concept of 3rd and 4th TEA tasks are similar, while the difference is in the task modality. The reliability and validity of the TEA were established in healthy, neurological, and psychiatric populations.

#### Brain engagement index

EEG data were recorded using the NeuroSky MindWave single-channel, wireless system (NeuroSky, Inc., San Jose, CA, CE-authorized), with one frontal dry electrode (∼Fpz) and one reference dry electrode on the earlobe, using a sampling rate of 512 Hz. The EEG data of continuous brain activity, sampled every 10 s, was used to extract an electrophysiological marker of sustained attention, called the BEI (Shahaf et al., 2017). Each 10-s segment was filtered to the delta band and then divided into epochs of 1,500 ms with overlapping. The power of delta activity was computed, and then the mean and standard deviation of all epochs within a segment were calculated. Epochs were regarded as noisy if the ratio of the standard deviation to the mean of absolute activity was greater than 1, then these 1,500 ms epochs were rejected and not included in the BEI computation. Ten-second segments with multiple rejected 1,500-ms epochs were automatically rejected, and a minimum of 3 consecutive 10-s segments were required for a valid sample. BEI computation is based on a template matching algorithm that compares a template of 1,500-ms attention-related delta bandpass activity with the ongoing sampled signal using a moving window (for a full description of the matching procedure, see Shahaf et al., 2017). Simply put, BEI is based on measuring the number of occurrences of delta pattern, the template, which is composed of a sequence of larger wave deflections, lasting a few hundred milliseconds, followed by a sequence of smaller waves, also lasting a few hundred milliseconds. The BEI is represented on a scale of 0–1. The value representing the optimal attentional allocation is 0.5 with a normal range between 0.3 and 0.7. BEI is presented ongoingly on the computer screen and might be used during clinical intervention and rehabilitation. The feasibility and validity of the BEI marker for attention were shown in previous research on stroke (Bartur et al., 2017), migraine (Shahaf et al., 2018b), depression (Shahaf et al., 2017), and ADHD (Shahaf et al., 2018b).

### Procedure

The study was approved by the Ethics Committee of Tel Aviv University and data was collected in 2018. All participants were volunteers who weren’t paid for their participation and were recruited through social networks. They provided written informed consent after receiving an explanation of the study’s aims and procedures. The study was carried out in a laboratory setting with constant conditions as for the light, noise, temperature, etc. All the participants sat in front of a desk with the study materials. First, the participants completed the demographic questionnaire and the MoCA test (no obligate certification was needed) to ensure inclusion and rule out exclusion criteria. Next, the EEG electrode was positioned on the forehead and was activated in advance to ensure proper signal detection after stabilization. The participants performed a series of tests including TMT A and B and the TEA tasks (A to D) in a continuous fashion while monitoring ongoing EEG activity. The order of TMT and TEA was counterbalanced across participants and the order of the TEA subtests was constant to enable a gradual increase in task complexity. The entire procedure took approximately 1 h to be completed.

### Data processing

#### Outliers detection

We tested a model for unsupervised anomaly detection based on the BEI time series data of each task (10 trials). We used a neighbor-based algorithm, Local Outlier Factor (Sklearn in Pinguin), with the Dynamic Time Warping (DTW) similarity metric (tslearn). The Local Outlier Factor is a statistical method that can identify anomalies based on statistical measures extracted from the data, thereby producing an outlier score for each trial based on the local density of the data. The algorithm’s continuous scores (outlier scores) were taken as a dependent variable to test for group differences (outliers, normal) in terms of performance on the TEA and TMT tasks. Following an exploration of the hyper-parameter space, we choose the threshold that yielded the best performance of the method on the data of each task.

#### Distance from optimal brain engagement index score

We tested the relationship between successful trials and their corresponding EEG concentration signal. We took the absolute distance of EEG’s concentration signals from 0.5 (the halfway point) as a dependent parameter, and compared these scores on correct trials, to the scores of incorrect trials.

### Statistical analysis

Mean correct responses (Accuracy) and mean BEI (BEI) were calculated for each task. Mean BEI was also calculated for TMT-A and TMT-B. Accuracy and mean BEI on the TEA tasks were tested using Repeated Measures ANOVA with Tukey *Post hoc* analysis. Analysis of Covariance (ANCOVA) was used to test for group differences in performance based on the algorithm classifying participants into outlier or normal groups. One-way ANOVA was used to test for differences in distance from optimal BEI score between correct and incorrect trials. Pearson correlational analysis was used to test for an association between BEI and performance in TEA tasks, TMT, and in between. The level of significance was set to 0.05. All tests were performed using SPSS22 (Armonk, NY: IBM Corp.).

## Results

### Performance on the test of everyday attention tasks

Repeated Measures ANOVA with TASK as a within-subject factor was performed. The main effect of TASK was highly significant (*F* = 16.601, *p* < 0.001, η*p*^2^ = 0.293). *Post hoc* tests revealed that performance on the different tasks significantly differed from each other (TEA A from TEA B *p* < 0.001, TEA A from TEA C *p* = 0.038, TEA A from TEA D *p* < 0.001, TEA B from TEA C *p* = 0.001, TEA C from TEA D *p* < 0.001) aside from the difference between TEA B and TEA D which approached significance (*p* = 0.063).

### Brain engagement index on the test of everyday attention tasks

Repeated Measures ANOVA with TASK as a within-subject factor yielded a significant result (*F* = 3.129, *p* = 0.028, η*p*^2^ = 0.073). *Post hoc* tests revealed that BEI on TEA C significantly differed from BEI on TEA A (*p* = 0.000) and BEI on TEA B (*p* = 0.032) and approached significance on TEA D (*p* = 0.073).

### Correlational analyses

Accuracy on the visual task with manipulation (TEA C) significantly correlated with overall latency on TMT-A (*r* = 0.467, *p* = 0.002) and TMT-B (*r* = 313, *p* = 0.047). In addition, BEI on this task (TEA C) significantly correlated with overall latency on TMT-A (*r* = 0.317, *p* = 0.043) and TMT-B (*r* = 425, *p* = 0.006).

### Outliers detection

Following the results of performance and mean BEI analyses, we tested a model for anomaly detection based on the BEI time series data of each task (10 trials). As shown in the analysis of BEI as a function of task, BEI on the visual task (TEA C) was significantly different from the auditory tasks and contained more variability. The outlier score presented as a negative score. Groups (outliers, normal) were created based on a threshold score of –1.2 to maintain plausible group sizes (N in the outliers group = 8). ANCOVA with Group as between-subjects factor and age as a covariate was performed for performance on TEA C, TMT-A, and TMT-B. Significant main effect of GROUP was found for TMT-A only (*F* = 6.54, *p* = 0.015, η*p*^2^ = 0.154) with increased latency for outliers (M outliers = 34.625, M normal = 25.71) (Figure 3).

**FIGURE 1:**
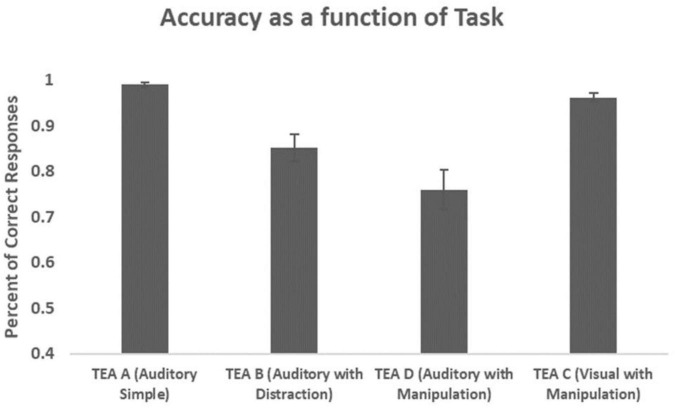
Accuracy as a function of task. Percents of correct responses are presented in bars (Mean, SE) for three auditory tasks (TEA A, TEA B, and TEA D) and one visual task (TEA C).

**FIGURE 2:**
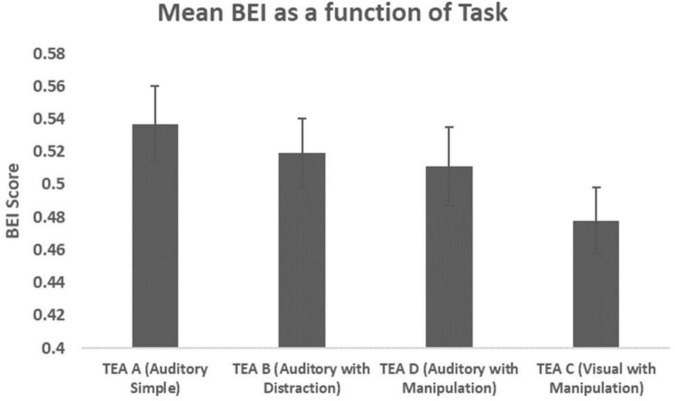
Mean BEI as a function of task. BEI scores are presented in bars (Mean, SE) for three auditory tasks (TEA A, TEA B, and TEA D) and one visual task (TEA C).

**FIGURE 3:**
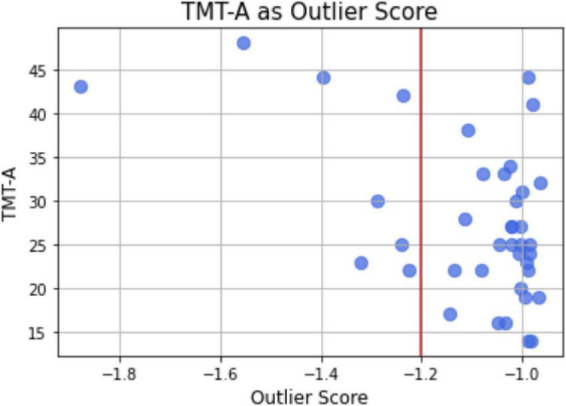
TMT-A as outlier score. Outliers detection model. scatter plot of outlier scores and TMT-A overall latency with a threshold of –1.2 to determine the two groups.

### Distance from optimal brain engagement index score

**FIGURE 4:**
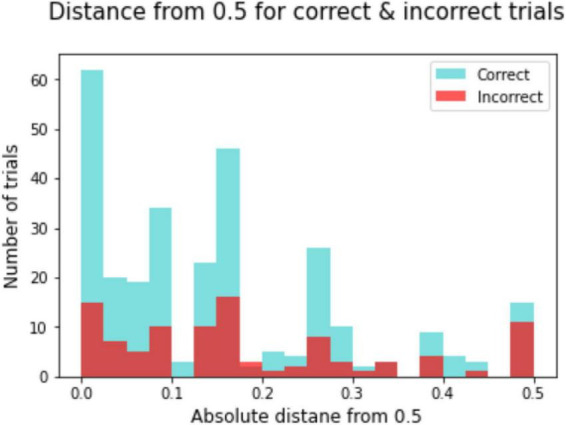
Distance from 0.5 for correct trials. Distance from Optimal BEI score. Number of correct and incorrect trials on the TEA C task across participants as a function of absolute distance (absolute) from the optimal BEI score of 0.5.

Distance from the optimal BEI score (0.5) is presented as absolute distance. Correct trials (*n* = 290) and incorrect trials (*n* = 100) were divided into two conditions. One-way ANOVA with CONDITION yielded a significant main effect of CONDITION for TEA D only (*F* = 4.520, *p* = 0.034, η*p*^2^ = 0.012) with correct trials in more proximate distance from the optimal BEI score (*M* = 0.149, *SD* = 0.137) than incorrect trials (*M* = 0.183, *SD* = 0.152) (Figure 4).

## Discussion

This pilot study was designed to investigate the feasibility of applying a single-channel, real-time, wireless EEG index of attention (BEI) during ecological tasks to explore patterns of sustained attention. First, we demonstrated that sustained attention is affected by task modality and task complexity in line with previous findings (Petersen and Posner, 2012). Specifically, we found a descending pattern of performance and a descending pattern of BEI through three auditory ecological tasks going from a simple task (TEA A) to a task with higher complexity due to distraction (TEA B), to a task with even higher complexity due to distraction and manipulation (TEA D). The observed descending linear patterns of performance and BEI across time and as a function of an increase in task complexity are similar to the results previously reported by studies using monotonous tasks such as CPT (Hahn et al., 2012; Esterman and Rothlein, 2019).

A different pattern was observed in the visual task with manipulation (TEA C), further supporting the impact of task modality on sustained attention (Szalma et al., 2004). Participants were expected to perform this task worse or similar to the auditory task with manipulation (TEA D) and to show a decrease in performance compared to the simple task (TEA A) and the task with distraction (TEA B). Interestingly, we found that performance on the complex visual task was better than on the similar auditory task (TEA D) and the simple auditory task with distractors (TEA B).

Contrary to an increase in performance, BEI significantly decreased in the visual task with manipulation (TEA C). This might indicate that participants required less attentional resources to efficiently perform the task. A possible reason for this finding is that the visual task is self-paced since there is no demand for the participants to respond in a restricted time window and time is measured overall. As such, the use of an ongoing EEG index of attention helped to reveal a more comprehensive pattern of sustained attention as a result of task intensity that could not be detected by performance alone. This emphasizes the importance of investigating cognitive functions in a naturalistic setting where participants interact with the environment without much constraint.

Performance on the visual task with manipulation (TEA C) was also found to positively correlate with time on the Trail Making Test. This finding may be understood based on the design of the two tasks and it is in line with previous findings (van der Leeuw et al., 2017). The Trail Making Test (TMT A and B) is a well-established neurocognitive task that requires visual screening and sequencing (A) and cognitive flexibility and switching, as part of attentional control (B). Likewise, successful performance of the TEA visual task with manipulation requires both (1) attentional switching with flexibility and (2) sequence maintenance. Moreover, the correlations between BEI during the performance of the visual task with manipulation (TEA C) and TMT (A & B) might reveal a linear association between behavioral and brain indices.

A model of anomaly detection further revealed that participants that were outliers in their pattern of electrophysiological activity during the visual task with manipulation (TEA C) were significantly slower on the TMT-A task. Both types of visual tasks, TMT and TEA C assess processing speed intending time to completion in addition to success rate. The compatibility between the tasks in terms of requirement might lead to the activation of similar mechanisms. Although we present preliminary findings, we suggest that this type of model for anomaly detection could be used to mark abnormal dynamics of brain activity during cognitive tasks. Indeed, Zhang et al. (2021) recently applied a classification model to monitor changes in attention states based on continuous measuring with a similar single-channel EEG device during gradCPT, which allows attention labeling.

The interplay between underlying brain activity and behavior is still debated. In fact, some posit that the traditional view assuming a binary division between health and pathology and a linear relation between neural activity and mental functions is incomplete (Northoff and Tumati, 2019). Recently, the Cognitive Effort Index (CEI) was used to dynamically identify the affective and cognitive barriers and the effort to achieve effective performance (Gillen and Shahaf, 2021); the authors proposed a complex graduated view of the dynamics of brain resources in relation to goals and difficulties. In the current study, we explored a simplified model showing that on successful trials in the auditory task with manipulation (TEA D) BEI was closer to the optimal value of 0.5. Although this model could be tested only on tasks in which there is enough variability of success rate among participants, it suggested that successful trials generally require more optimal brain activity.

The scope of the current findings supports the premise that there is no unitary theory for human performance since we observed different patterns of behavior and brain activity as a function of task modality and task complexity. The observed utility of implementing a one-channel wireless EEG index (BEI) for the investigation of behavior in ecological settings is congruent with previous research which demonstrated the feasibility and utility of wireless EEG monitoring in a variety of contexts such as for assessing attentiveness to instruction in class (Liu et al., 2013), driver’s fatigue (Ko et al., 2015), mental engagement of students during learning tasks (Khedher et al., 2019), and health state in intensive care units (Caricato et al., 2020). So, combined behavior and electrophysiological markers promote an innovative hands-on methodology to monitor behavior on a sub-second scale, detect outliers and predict important changes, and finally, personalize diagnosis and treatment.

The usage of consumer wireless EEG might require a different type of research than the typical EEG studies performed in the lab with high-density EEG devices. These costly high-density EEG devices collect high-quality recordings which could be later cleaned and processed in ways that are unavailable to single-channel devices, allowing high temporal resolution and even spatial specificity (e.g., source localization). However, studies using these devices are mostly conducted with a small number of participants in a restricted setting with very specific stimuli and their data is lengthily processed offline. Reducing attention-related brain activity to a recording from a single frontal channel is based on the premises that (1) it is possible to detect responsiveness from very few electrodes, even a single electrode (Shahaf et al., 2015), and (2) attentional functions are supported by spread networks with prefrontal involvement (Clayton et al., 2015). These premises allow the shift from EEG as a tool for investigating systems of attention to a simple electrophysiological signal, similar to other physiological signals used to monitor states such as in the case of anxiety.

Understanding fluctuations in sustained attention through different modalities and the extent of their demands is critical for everyday functioning. For example, while driving, we process up to 90% of the information visually, thus, successful driving requires proper functioning of visual sustained attention (Ojsteršek and Topolšek, 2019). On the other hand, academic learning is based on both visual and auditory sustained attention, being a highly demanding task in both modalities. Indeed, while the recommended interval of ongoing driving is 2 h (Chen and Xie, 2014), the span of academic learning is 30 min (Dunlosky et al., 2013). Moreover, these findings may further support the notion that there are differences in sustained attention functions in everyday tasks depending on the task modality with attentive drivers may be inattentive learners and vice versa.

Altogether, the current study reveals patterns of sustained attention during ecological tasks through behavioral and electrophysiological indices as a function of task modality and complexity. Moreover, applying models to detect outliers and optimal range of attention were the first steps in exploring the practical use of such an electrophysiological index (BEI). The study nonetheless has several limitations. First, given the EEG prefrontal recording, there is always concern regarding the contamination of the EEG signal with noise coming from muscles and eye movements (EMG and EOG). However, eye blink rate and frontal muscle activity are considered by some researchers to be an index of attention and therefore are not necessarily removed in line with traditional methods of artifact rejection (Maffei and Angrilli, 2018). Future studies are encouraged to combine additional measurements such as EOG and EMG. Regarding the principal task, only the auditory task was graduated through three separated subtests, while the visual task was graduated during the trials of the visual subtest. Even though this was dictated by the design of the TEA, the only available ecological test of attention for the target population, this methodological issue may prevent a full comparison between modalities. We recommended further development and validation of ecological attentional tasks to enable in-depth investigation of the effects of task complexity. Additionally, the subtests of the TEA were administered in a fixed order, limiting our ability to conclude the possible confounds between task complexity and time on task. Important limitations of the study are related to the participants due to convenience sampling which restricts large-scale generalization of the findings, inequitable representation of sexes with 68% females, and the need for a higher cutoff score for the MoCA as well as other methods to ensure intact cognitive functioning. Further research is needed to explore the implementation of BEI in larger groups, in different populations, and using various cognitive tasks and setups to deepen our understanding of failures in sustained attention in real life. Finally, other models compatible with continuous measurement in ecological settings should be tested to advance the personalization of neurocognitive diagnosis and intervention.

## Data availability statement

The raw data supporting the conclusions of this article will be made available by the authors, without undue reservation.

## Ethics statement

The studies involving human participants were reviewed and approved by the Tel Aviv University Ethic Committee. The patients/participants provided their written informed consent to participate in this study.

## Author contributions

LL-V conceptualized and designed the study. KA was responsible for the data interpretation. NG was responsible for the data analysis. RK carried out the data collection. All authors actively involved in different stages of the manuscript preparation.
